# A selective electrochemical sensor for caffeic acid and photocatalyst for metronidazole drug pollutant - A dual role by rod-like SrV_2_O_6_

**DOI:** 10.1038/s41598-017-07423-1

**Published:** 2017-08-03

**Authors:** R. Karthik, J. Vinoth Kumar, Shen-Ming Chen, P. Senthil Kumar, V. Selvam, V. Muthuraj

**Affiliations:** 1 Department of Chemical Engineering, National Taipei University of Technology, No. 1, Section 3, Chung-Hsiao East Road, Taipei, ROC 106 Taiwan; 2 Department of Chemistry, VHNSN College, Virudhunagar, 626001 Tamilnadu India; 3 Chemistry of Heterocycles & Natural Product Research Laboratory, Department of Chemistry, School of Advanced Sciences, VIT University, 632014 Vellore, Tamilnadu India

## Abstract

In the present study, well-defined one-dimensional (1D) rod-like strontium vanadate (SrV_2_O_6_) was prepared by simple hydrothermal method without using any other surfactants/templates. The successful formation of rod-like SrV_2_O_6_ was confirmed by various analytical and spectroscopic techniques. Interestingly, for the first time the dual role of as-prepared rod-like SrV_2_O_6_ were employed as an electrochemical sensor for the detection of caffeic acid (CA) as well as visible light active photocatalyst for the degradation of metronidazole (MNZ) antibiotic drug. As an electrochemical sensor, the SrV_2_O_6_ modified glassy carbon electrode (GCE) demonstrated a superior electrocatalytic activity for the detection of CA by chronoamperometry and cyclic voltammetry (CVs). In addition, the electrochemical sensor exhibited a good current response for CA with excellent selectivity, wide linear response range, lower detection limit and sensitivity of 0.01–207 µM, 4 nM and 2.064 μA μM^−1^cm^−2^, respectively. On the other hand, as-synthesized rod-like SrV_2_O_6_ showed highly efficient and versatile photocatalytic performances for the degradation of MNZ, which degrades above 98% of MNZ solution under visible light irradiation within 60 min. The obtained results evidenced that the improvement of rod-like SrV_2_O_6_ might be a resourceful electrocatalyst and photocatalyst material in the probable applications of environmental and biomedical applications.

## Introduction

In the past few decades, polyphenols are paid great attention in biological, chemical and nutrient fields owing to their extraordinary antioxidant, anti-inflammatory and anti-microbial properties^1, 2^. Caffeic acid (CA) is one of the polyphenol derivatives which occur in coffee, olive oil, propolis, some fresh vegetables, fruits, cosmetics and red wines^3^. In CA structure there is two hydroxyl groups is presented, this two hydroxyl groups is significantly contributed to the unique antioxidant properties^4^. Moreover, CA plays a vital role in human life circle process including the treatment of asthma, immune regulation diseases, allergic reactions, atherosclerosis and other cardiovascular diseases^5^. Due to its peculiar anti-oxidant properties, it can be act as a carcinogenic inhibitor and also prevents the replication of HIV/AIDS^6, 7^. In addition, 3 µg of CA can be used as a drug for the treatment of snake poisoning as well as 100 µg of CA can prevent the venous endothelial cells. However, the suitable dosage of CA is listed as 0.3–0.9 g per day and over dosage may causes negative impact to the human beings^8^. Therefore, the accurate level of CA detection is an important phenomenon to the environment and pharmaceutical formulations. Up to now, different analytical techniques such as chromatography, spectrophotometry, UV-vis spectrophotometry, capillary electrophoresis^9–12^ and electrochemical sensor have been developed for the detection of CA. Among all, the electrochemical techniques are more adoptable due to its simplicity, low-cost, fast response, practicality, high sensitivity and excellent selectivity^13–15^.

On the other hand, the release of pharmaceutical wastes such as antibiotics, analgesics, antipyretics, hormones and antimicrobials release into the water and solid environment can causes negative impact to the living things. In specific, metronidazole (MNZ) antibiotic drug which is widely used in the treatment of protozoa, amoebiasis, trichomoniasis and giardiasis^16, 17^. In addition, MNZ has also been used as an additive in poultry and fish feed industries to remove parasites^18^. The long-term release of MNZ into the environment can generates carcinogenic and mutagenic diseases to the living things^19^. However, MNZ has high solubility in water, non-biodegradability and faintly adsorbed on soil environment^20, 21^. For that reason, the removal of MNZ into the aquatic and soil environment is an important concern to prevent the living organisms. Various techniques have been proposed for the removal of MNZ into the environment including adsorption, biological treatments, ozonation, Fenton, photo-Fenton process and photolysis^22^. For aforementioned techniques, photocatalysis could offers simple, low-cost and eco-friendly technique for the removal of MNZ into the environment^23–33^.

Recently, transition metal based vanadates have much attention to the researchers owing to their low-cost, non-toxic in nature and which has been widely used in various potential applications^34^. In particularly, strontium based vanadates has considerable attention due to their optical, and electrical properties which can be used as an variety of applications including gas sensors, solid state electrolytes, lithium-ion batteries, electrochemical sensor and photocatalyst^35, 36^. There are several types of mixed oxides based on strontium and vanadium such as SrVO_3_, Sr_3_V_2_O_8_, Sr_2_V_2_O_7_, Sr_3_(VO_4_)_2_ and SrV_2_O_6_ have been reported^37–40^. However, the electrochemical and photocatalytic properties of SrV_2_O_6_ phase reports are scarce until now. Recently, Z. Li *et al*., reported dandelion-like micro-crystallites and long belt-like nanostructures of SrV_2_O_6_ phase synthesized by hydrothermal method in the presence of adipic acid^40^. It is well known that the interesting photocatalytic and electrochemical behaviors mainly dependent on its morphologies and crystalline nature^41, 42^. In specifically, low-dimensional nanomaterials such as nanotubes, nanowires, nanoribbons and nanorods have been focused on the concentrated research due to their peculiar properties and its applications in physics and construction of nanoscale devices^43^. Hence, it is extremely imperative to build up a simple and rapid synthesis procedure for the preparation of SrV_2_O_6_ phase with one-dimensional structure as well as high crystallinity under eco-friendly nature.

Herein, we made attempt to synthesize rod-like SrV_2_O_6_ through simple hydrothermal treatment followed by the annealing process. The prepared rod-like SrV_2_O_6_ was characterized by using X-ray diffraction (XRD), transmission electron microscopy (TEM), scanning electron microscopy (SEM), energy-dispersive X-ray (EDX), UV-Vis diffuse reflectance (UV-DRS), Raman and X-ray photoelectron spectroscopy (XPS). Fascinatingly, the rod-like SrV_2_O_6_ displayed a greatly energetic catalyst for the electrochemical determination of CA as well as, it was an efficient visible light photocatalyst for the degradation of MNZ antibiotic in the environmental samples that were briefly investigated with high degradation rate.

## Result and Discussion

### Characterization of the rod-like SrV_2_O_6_

**Figure 1:**
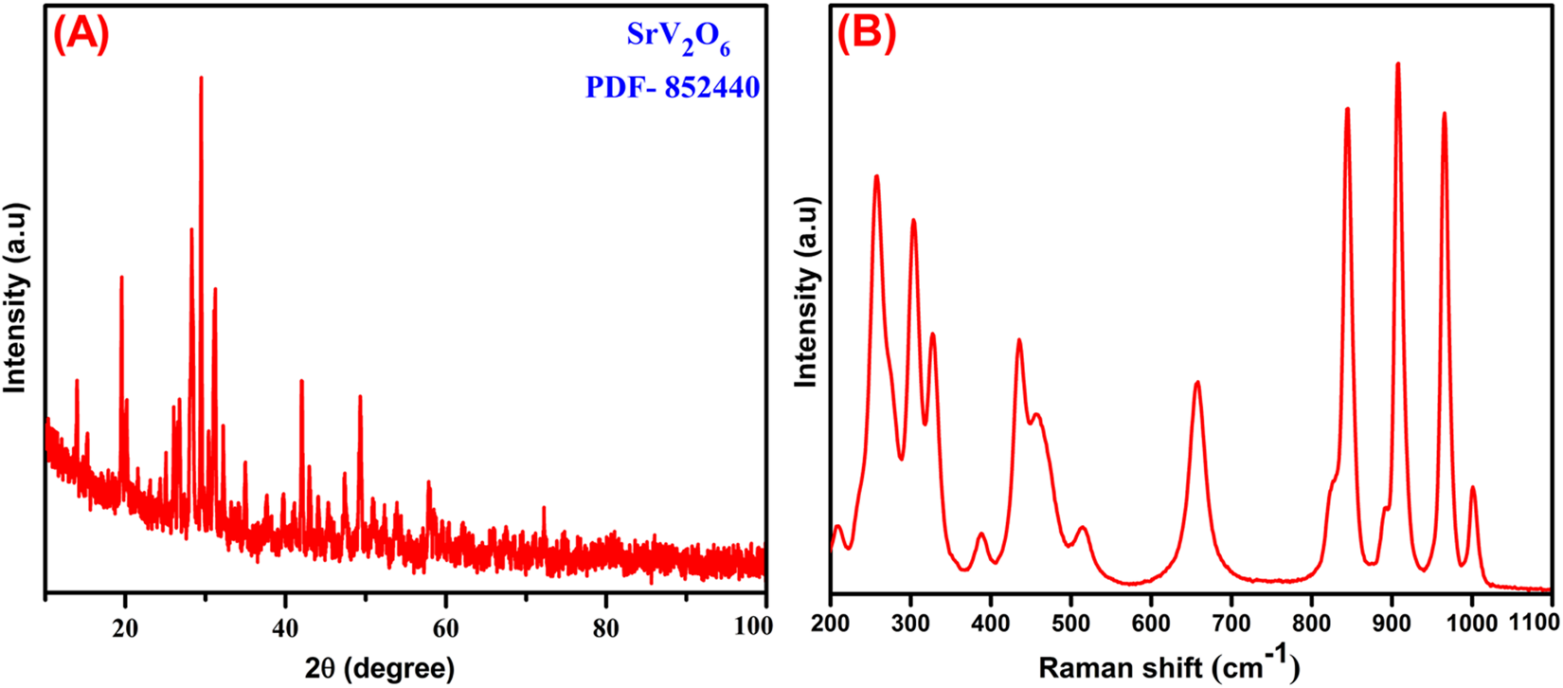
XRD pattern of SrV_2_O_6_ (**A**) and Raman spectrum of SrV_2_O_6_ (**B**).

The crystal structure and phase purity of as-prepared SrV_2_O_6_ were investigated by using XRD analysis as it shown in Fig. 1(A). From the Fig. 1(A), all the intense diffraction peaks can be indexed to the facets of orthorhombic phase SrV_2_O_6_ and well matched with Joint Committee on Powder Diffraction Standards (JCPDS) data **[No**. **852440]**. The calculated lattice parameters are a=9.666, b = 3.680, c = 12.529 Å, with the space group of Pnma. No other extra peaks were detected related to the SrO_2_ and V_2_O_5_, which suggested that high phase purity and the sharp peaks revealed the good crystalline nature of SrV_2_O_6_. From the XRD results, the as-synthesized SrV_2_O_6_ is in β-SrV_2_O_6_^40^. Raman spectroscopy is an effective technique to analyze the bonding states and local structure of the as-synthesized rod-like SrV_2_O_6_ as it shown in Fig. 1(B). The obtained Raman bands below in the region of 400 and 434 cm^−1^ which belongs to the O-V-O twisting, rocking and chain deformation vibrations, and δ-VO_2_ vibrations of SrV_2_O_6_, respectively^44^. The bands at 514 and 655 cm^−1^ attributed to the V-O-V symmetric stretching vibrations. The sharp and high intensity bands in the region of 800–1000 cm^−1^ were assigned to the VO_2_ stretching vibrations^45^.

**Figure 2:**
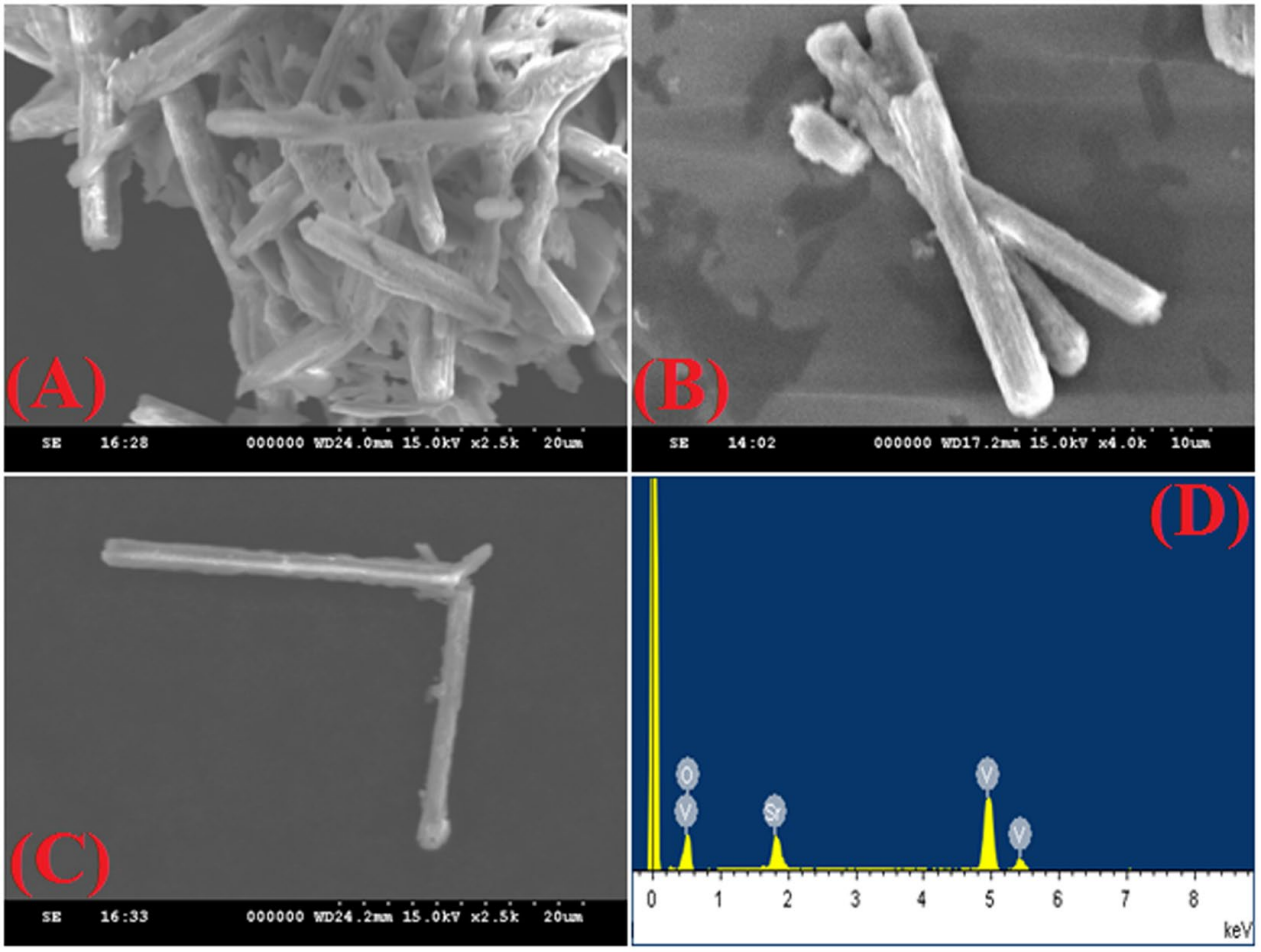
SEM images of SrV_2_O_6_ at different magnification 20 µm (**A-C**) 10 µm (**B**) and corresponding to EDX spectrum of SrV_2_O_6_ (**D**).

**Figure 3:**
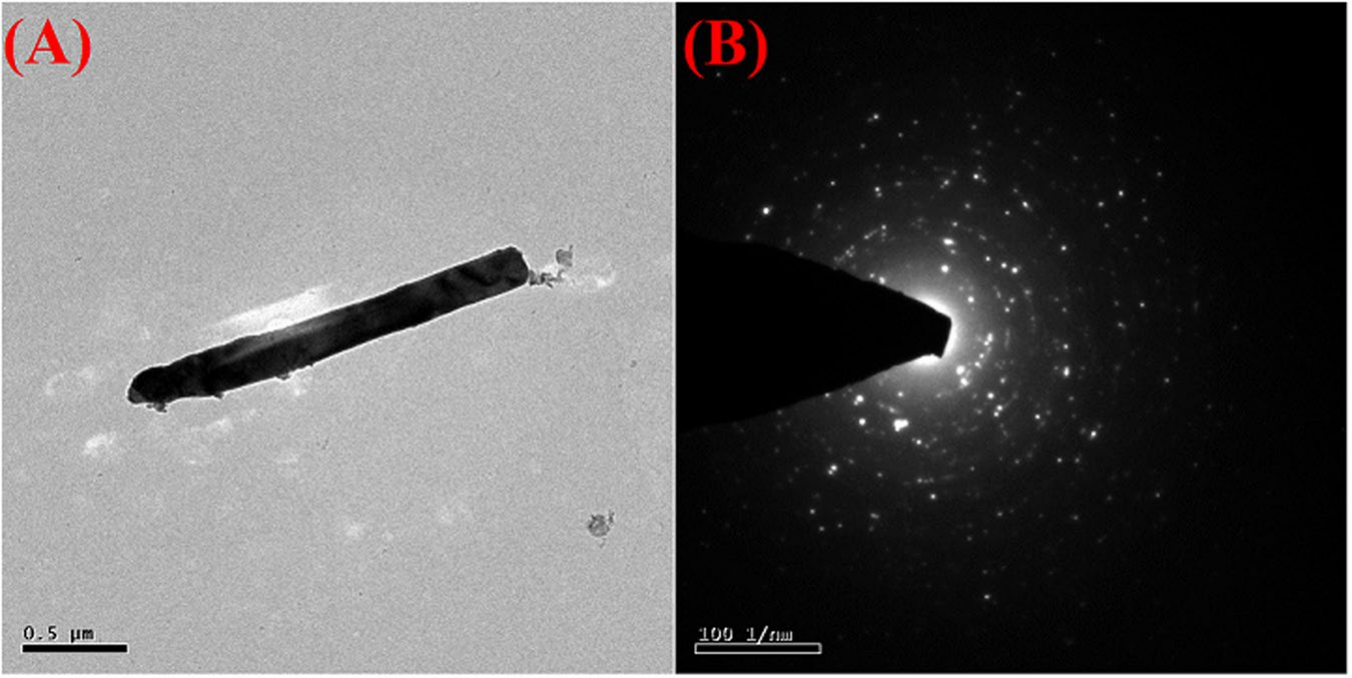
TEM image of SrV_2_O_6_ (**A**) and SAED spectrum of SrV_2_O_6_ (**B**).

The surface morphology of the as-synthesized SrV_2_O_6_ were observed by SEM analysis as shown in Fig. 2(A). The different magnification of the SEM images (Fig. 2A-C) revealed the presence of large quantity of rod-like structures that is closely arranged with each other. The high magnification SEM images displayed smooth and well defined rod-like morphology and the average diameter is about 90–100 nm. To study the elemental composition, the prepared rod-like SrV_2_O_6_ were investigated by using EDX. Figure 2D revealed that the formed rod-like SrV_2_O_6_ were composed of strontium, vanadium and oxygen, at the same time no other signals was observed in the EDX spectrum which suggested that the purity of the as-prepared rod-like SrV_2_O_6_. The detailed surface morphology and size was further evidenced by TEM analysis as shown in Fig. 3. The TEM image clearly confirmed that the formation of rod-like structure with in the micro-range (Fig. 3A) and also discloses well defined boundaries.

The selected area electron diffraction (SAED) pattern (Fig. 3B) apparently visualized that the bright spots which is corresponds to the different orientation planes of SrV_2_O_6_ and it was further confirmation for the high crystalline nature. Furthermore, the elemental composition and their distributions were strongly evidenced by elemental mapping analysis as it can be seen in Fig. S1. Elemental mapping portrays the coexistence and uniform distribution of Sr, V and O elements in the rod-like SrV_2_O_6_.

**Figure 4:**
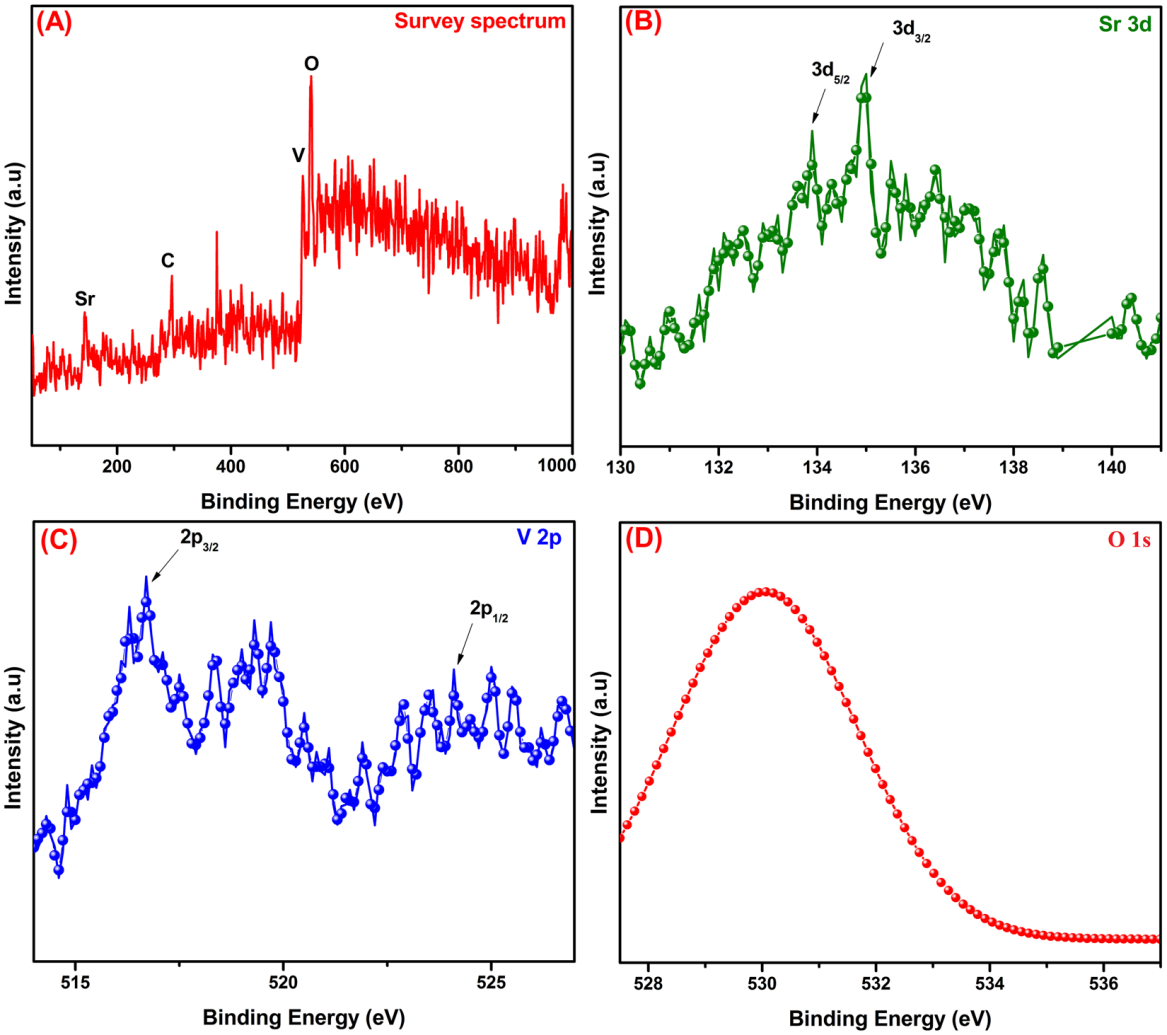
XPS survey spectra of SrV_2_O_6_ (**A**),) High resolution XPS spectra of Sr 3d, V 2p and O 1 s (**B**–**D**).

Furthermore, the surface composition and their oxidation states were accurately determined by XPS analysis as shown in Fig. 4. The overall XPS survey spectrum (Fig. 4A) displays the presence of Sr, C, V and O elements which is good consistence with EDX report. The presence of C at 284.5 eV might be attributed to the residual hydrocarbon from the XPS instrument. The high-resolution XPS spectrum of Sr delivers the peaks at the binding energies of 133.4 and 134.9 eV, which corresponds to the Sr 3d_5/2_ and Sr 3d_3/2_ (Fig. 4B) state respectively, suggesting the existence of Sr^2+^ oxidation state^46^. From the Fig. 4C, the intense peaks at 517.2 and 524.08 eV are related to the binding energies of V 2p_3/2_ and V 2p_1/2_ spin-orbits, implying that the V^5+^ oxidation state^47^. In the enlarged O 1 s spectrum shows the peaks in the range of 529.2 to 531.03 eV could be ascribed to the O^2−^ state in SrV_2_O_6_^48^.

**Figure 5:**
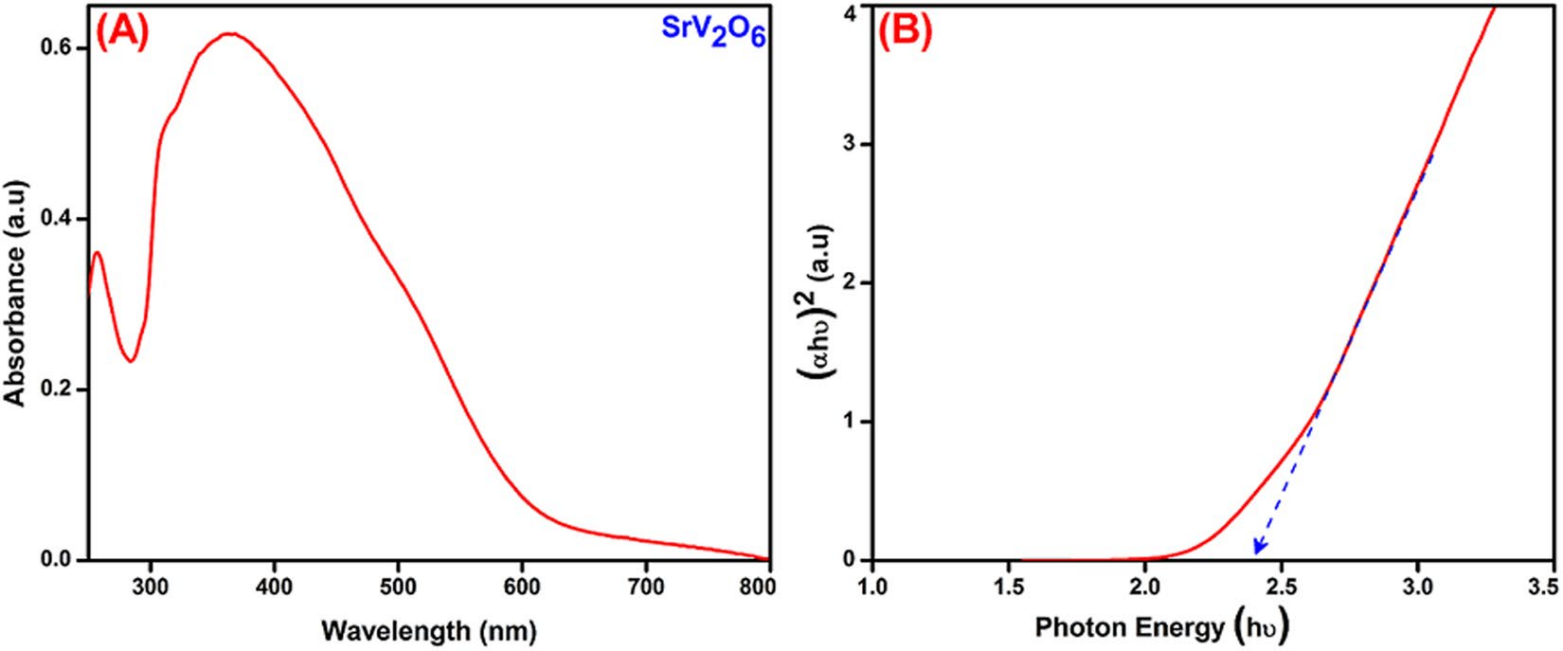
UV-Vis diffuse reflectance spectra (DRS) (**A**) and Energy gap spectra of rod-like SrV_2_O_6_ (**B**).

The optical properties and the active light region for the rod-like SrV_2_O_6_ were analyzed by UV-visible spectroscopy as illustrated in Fig. 5A. The spectrum exhibited a broad absorption peak from 320 to 550 nm which is corresponded to the bathochromic shift. It was clearly revealed that the rod-like SrV_2_O_6_ may active in the visible light region. However, to predict the exact wavelength required for the photocatalytic reaction, the energy gap was calculated using tauc’s equation and is shown in Fig. 5B. The energy gap calculated for the rod-like SrV_2_O_6_ is 2.4 eV.

**Figure 6:**
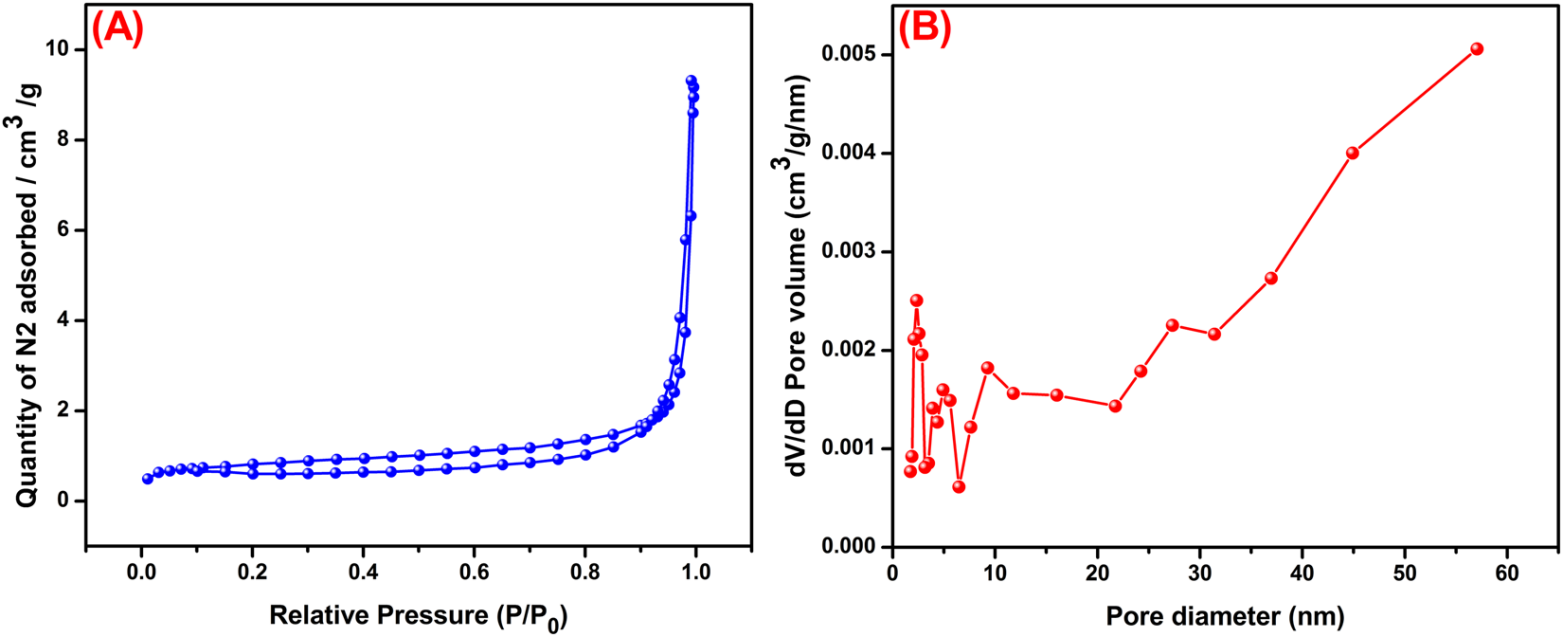
Nitrogen adsorption-desorption isotherms (**A**) and the pore size distribution plots (**B**) of rod-like SrV_2_O_6_.

The surface area of the catalyst act as significant role in the photodegradation process because of higher surface area of the catalyst can generates higher number of reactive species which results the higher photocatalytic activity of the catalyst^27^. The N_2_ adsorption-desorption isotherm analysis was used to identify the specific surface area and porous structure of as-synthesized rod-like SrV_2_O_6_ and shown in Fig. 6. From the Fig. 6A, the specific Brunauer-Emmett-Teller (BET) surface area of the rod-like SrV_2_O_6_ was determined to be 4.7 m^2^/g. The average pore-size distribution of rod-like SrV_2_O_6_ was derived from Barrett–Joyner–Halenda (BJH) pattern as represented in Fig. 6B. It obvious that rod-like SrV_2_O_6_ was contains mesoporous range of 26 nm.

### Electrochemical performance of CA at SrV_2_O_6_ modified GCE

**Figure 7:**
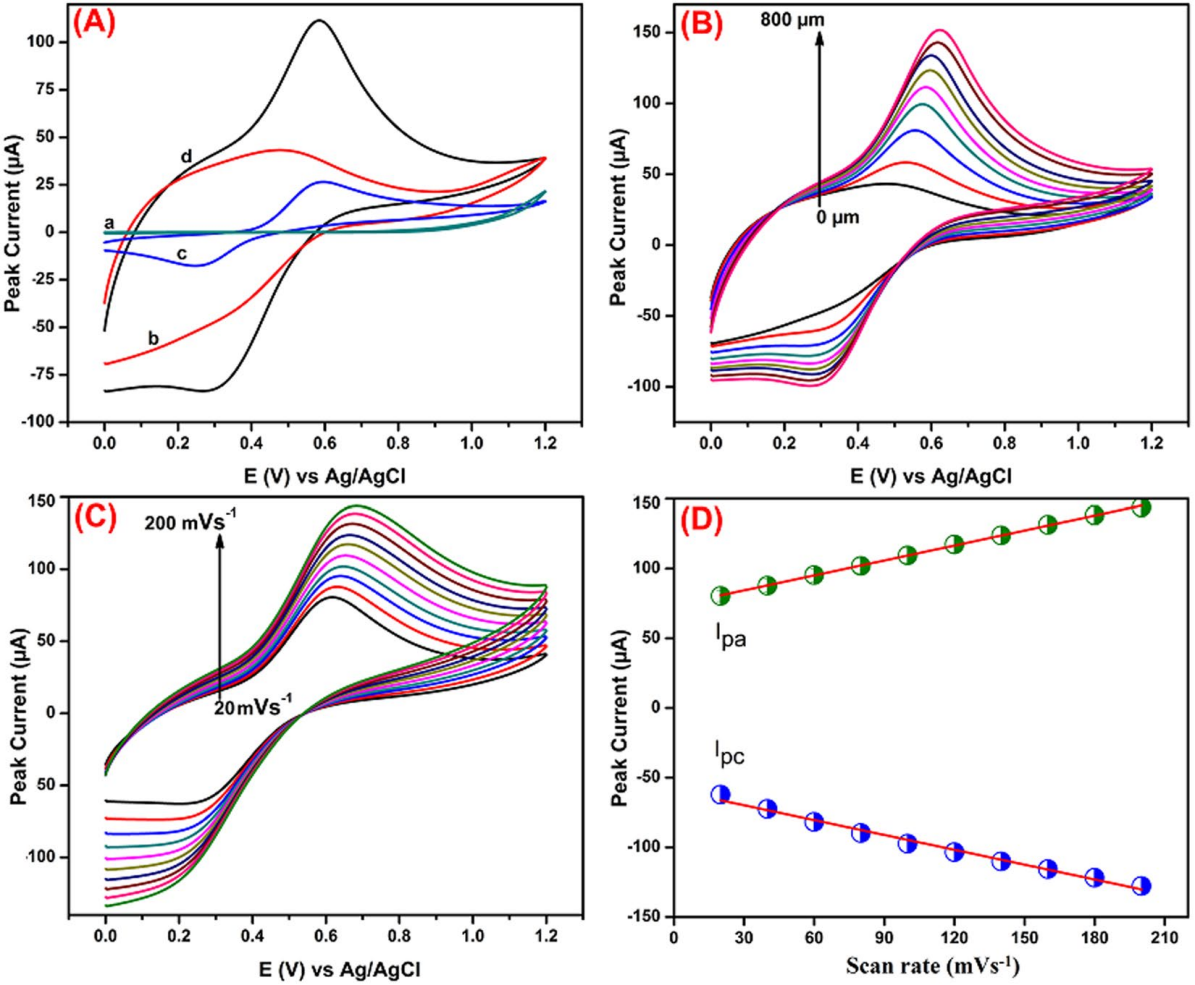
CVs performance of CA at different modified electrodes bare GCE (a) SrV_2_O_6_/GCE (b) in the presence (c,d) and absence (a,b) of 400 µM CA containing 0.1 M KCl at a scan rate 50 mVs^−1^ (**A**). CVs performance of CA for various concentrations from 0 to 800 µM (**B**). Different scan rates ranging from 20–200 mVs^−1^, 400 µM CA using SrV_2_O_6_/GCE in the 0.1 M KCl (**C**). The relation between the peak current *vs*. scan rate (**D**).

The electrochemical redox performance of CA was studied at various modified and unmodified GCE such as (a) bare GCE (b) SrV_2_O_6_/GCE by using CVs in the presence (c,d) and absence (a,b) of 400 µM CA containing 0.1 M KCl at a scan rate 50 mVs^−1^ (Fig. 7A). The potentials were scanned in the range from 0 to 1.2 V. From the Fig. 7A(a), the unmodified GCE didn’t shows any redox peak current at the potential range from 0 to 1.2 V, which demonstrates that the unmodified bare GCE is not suitable for the detection of CA. At the same time, the presence of CA (Fig. 7A(c,d)), a couple of redox peak was observed for the bare GCE and rod-like SrV_2_O_6_ modified GCE at the potential of 0.61 and 0.24 V, 0.57 and 0.27 V, respectively. Figure 7A(c) shows the a pair of weak redox peak was observed at the bare GCE with peak-to-peak separation of (Δ *E*p) 338 mV, which is clearly indicating that the redox peaks must me aspects to the electrochemical redox activities of CA. For the rod-like SrV_2_O_6_ modified GCE (Fig. 7A(d)), a pair of quasi-reversible redox behavior peak current considerably increased when compared with bare GCE, it’s due to the excellent conductivity and high specific surface area of rod-like SrV_2_O_6_. The peak-to-peak separation for CA at SrV_2_O_6_/GCE was found as 302 mV. The anodic peak current of CA at SrV_2_O_6_/GCE is 3.5 times much higher when compared to the bare GCE. The obtained results suggesting that the synthesized rod-like SrV_2_O_6_ possess good interaction ability towards the CA. So, we recommended that the rod-like SrV_2_O_6_ is very suitable electrode active material for the sensitive and selective determination of CA in future electrochemical sensor fabrication. On the other hand, increasing the concentrations of CA from 0 to 800 µM the redox peak current was increased gradually (Fig. 7B), which is indicating that the rod-like SrV_2_O_6_ possess high catalytic activity towards the CA. The sensitivity and the limit of detection (LOD) are discussed and detailed in the amperometric section.

### Effect of scan rate

The CVs for the redox of CA at the rod-like SrV_2_O_6_ modified GCE in 0.1 M KCl containing 400 µM CA as shown in Fig. 7C. There was a negative shift in the reduction peak potential and a positive shift in the oxidation peak potential with increase the scan rate from 20 to 200 mVs^−1^. The anodic and cathodic peak current of CA was also increased with increasing the scan rates from 20 to 200 mVs^−1^. The linear relationship was plotted between both anodic, cathodic peak currents and scan rates (Fig. 7D) and their linear regression equations can be expressed as follows: *I*_*pa*_ (µA) = 0.3575 + 73.7 (mVs^−1^) with correlation co-efficient R^2^ = 0.9993; *I*_*pc*_ (µA) = −0.3553–59.166 (mVs^−1^) (R^2^ = 0.9898) respectively, suggesting that the rod-like SrV_2_O_6_ modified electrode process of CA is a typical-adsorption controlled process.

### Determination of CA

**Figure 8:**
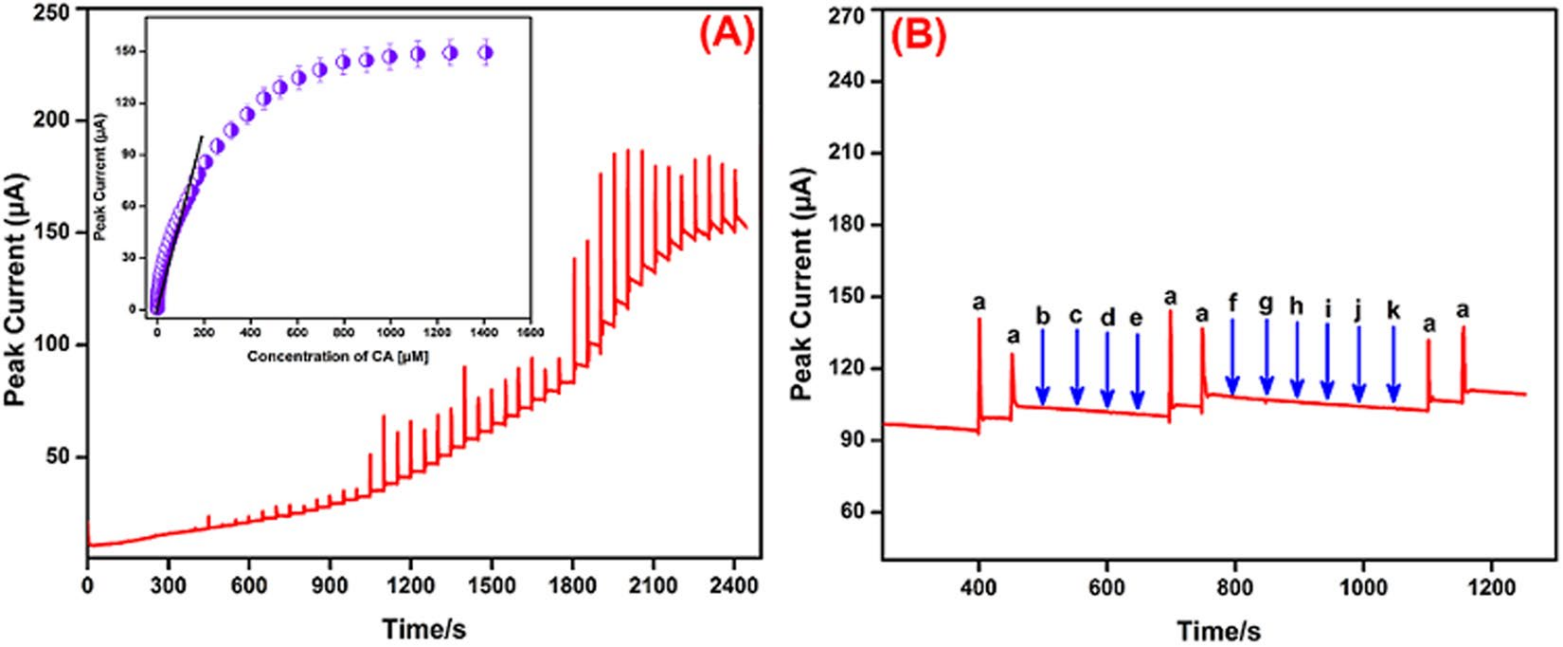
Amperometric (*i-t*) response at SrV_2_O_6_ modified RDGCE upon successive additions of 0.01–1407 µM CA into continuously stirred 0.1 M KCl. Applied potential + 0.58 V (**A**). At the same working conditions, amperometric response at SrV_2_O_6_ modified RDGCE for 50 µM of CA (a) 50 fold excess concentration of biologically co-interfering substances glucose (b), galactose (c), fructose (d), sucrose (e), catechol (f), ferulic acid (g), gallic acid (h), uric acid (i), ascorbic acid (j), dopamine (k) into continuously stirred 0.1 M KCl (**B**).

The amperometric (*i-t*) technique is a higher sensitivity and better resolution technique when compared with conventional CVs. In order to we used this amperometric technique for the determination of CA. Figure 8A shows the amperogram obtained at the rod-like SrV_2_O_6_ modified RDGCE towards the each continuous addition of CA (0.01- 1407 µM). Every addition was injected at the time intervals of 50 s into the continuously stirred 0.1 M KCl with the rotation speed at 1200 rpm. The applied constant working potential of the electrode was held at + 0.58 V. Figure 8A displays that rod-like SrV_2_O_6_ modified RDGCE shows a well-defined and rapid amperometric current responses was obtained in various addition of CA concentration with the response time of 5 s, which is clearly indicating that the quick electron movement process was happened in the electrode and electrolyte interface when injecting the CA. The peak currents was increased linearly with increasing the concentration of CA from lower to higher and the peak currents was also linearly increased and reached steady state current. A calibration plot was plotted for the CA concentration *vs*. anodic peak current (inset: Fig. 8A) and the linear regression equation can be expressed as *I*_*p*_ (µA) = 0.4128 [CA (µM)] + 10.713. The amperometric current responses were increased linearly with increasing the CA concentration in the linear range from 0.01 to 207 µM. The limit of detection (LOD) and sensitivity was calculated as 4 nM and 2.064

µAµM^−1^cm^−2^, respectively. The obtained analytical parameters such as linear range and LOD at rod-like SrV_2_O_6_ modified RDGCE towards the determination of CA have been compared with previously reported literature and are summarized in Table 1. From the literature results, suggesting that the proposed sensor exhibited good comparable analytical parameters with other reported electrochemical CA sensor. This should be attributed to the excellent electrocatalytic skill of the rod-like SrV_2_O_6_ modified RDGCE for the sensitive and selective electrochemical determination of CA.

**Table 1:** Comparison of the analytical performance for proposed method with other reported chemically modified electrochemical detection of CA.

Electrode	Linear range (µM)	LOD (µM)	Ref.
PG/GCE	9–40	3.9	49
Plant-peroxidase-chitin/CPE	20–200	2	50
Glassy polymeric carbon	0.9–11	0.2	5
PbFE	0.01–0.5	0.004	51
AuNPs -chitosan/GE	0.05–2000	0.025	52
AuNP/GN/GCE	0.05–50	0.05	53
Nafion/ER-GO/GCE	0.01–1.5	0.09	54
MIS/GE	0.5–60	0.15	55
LDHf/GCE	7–180	2.6	56
PGE	0.01–3000	0.08	57
MWNTs-[BuPy]PF_6_-CS/GCE	0.02–7	0.005	58
Laccase biosensor (LTV-SPE)	0.5–1300	0.52	59
Laccase-MWCNT-chitosan/Au	0.7–10	0.15	60
Tyrosinase/diazonium/SPGE	0.3–83	0.2	61
LuPc_2_ nanowires/ITO	60–5000	3.12	62
Nafion/Tyre/Sonogel-Carbon	0.08–2	0.06	63
Glassy polymeric carbon	96.5–0.1	0.29	64
Glassy carbon electrode	10–120	0.1	65
Rod-like SrV_2_O_6_/GCE	0.01–207	0.004	This work

Abbreviation: PG-Poly(glutamic acid; GCE-glassy carbon electrode; CPE-carbon paste electrode; PbFE-lead film electrode; AuNPs-colloidal gold nanoparticles; GE-gold electrode; GN-graphene Nanosheets; ER-GO-electrochemically reduced graphene oxide; MIS-molecularly imprinted siloxanes; LDHf-layered double hydroxide film; PGE-pencil graphite electrode; MWCNT- Multiwalled Carbon Nanotubes; [BuPy]PF_6_-Butylpyridinium Hexafluorophosphate; CS- Chitosan; LTV- Laccase (*Trametes versicolor*); SPE- Screen printed electrode; Au- Gold sheets electrode; SPGE- Screen printed gold electrode; LuPc_2_-Lutrium bisphthalocyanine; ITO- Indium tin oxie.

In order to investigate the selectivity of the rod-like SrV_2_O_6_ modified RDGCE towards the detection of CA in the presence of various potentially co-interfering substances by amperometric technique. As shown in Fig. 8B, the SrV_2_O_6_/RDGCE shows well-defined amperometric current responses towards the each addition of 50 µM of CA (a), at the same time, there is no noteworthy peak current was observed in the presence of 50 fold excess concentration of biologically co-interfering substances such as glucose (b), galactose (c), fructose (d), sucrose (e), catechol (f), ferulic acid (g), gallic acid (h), uric acid (i), ascorbic acid (j), dopamine (k) in a continuously stirred 0.1 M KCl. Further, 50 µM CA addition (a) produced same signal amperometric peak current responses in the presence of aforementioned interfering substances, suggesting that the proposed SrV_2_O_6_/RDGCE sensor possessed excellent selectivity. Therefore, it can be used as selective electrochemical sensor for the detection of CA even in the presence of more interfering substances.

### Stability, Reproducibility and Repeatability studies

The stability of the electrode is very important factor for the newly developed electrochemical sensor. To examine the stability of the SrV_2_O_6_ modified GCE up to 7 days by CVs and stored in 0.1 M KCl when not in use. The modified electrode retained 95.2% of its initial response peak current after 7 days, which suggesting that the SrV_2_O_6_ modified GCE had appreciable storage stability of the sensor. The reproducibility of the sensor was also investigated in 3 independent modified electrodes by CVs in the presence of 50 µM CA displays a acceptable reproducibility with the relative standard deviation (RSD) of 3.6%. Furthermore, the proposed electrochemical sensor offered adequate repeatability with an RSD of 3.2% for 10 consecutive repeated measurements in a single modified electrode.

### Photocatalytic performance

**Figure 9:**
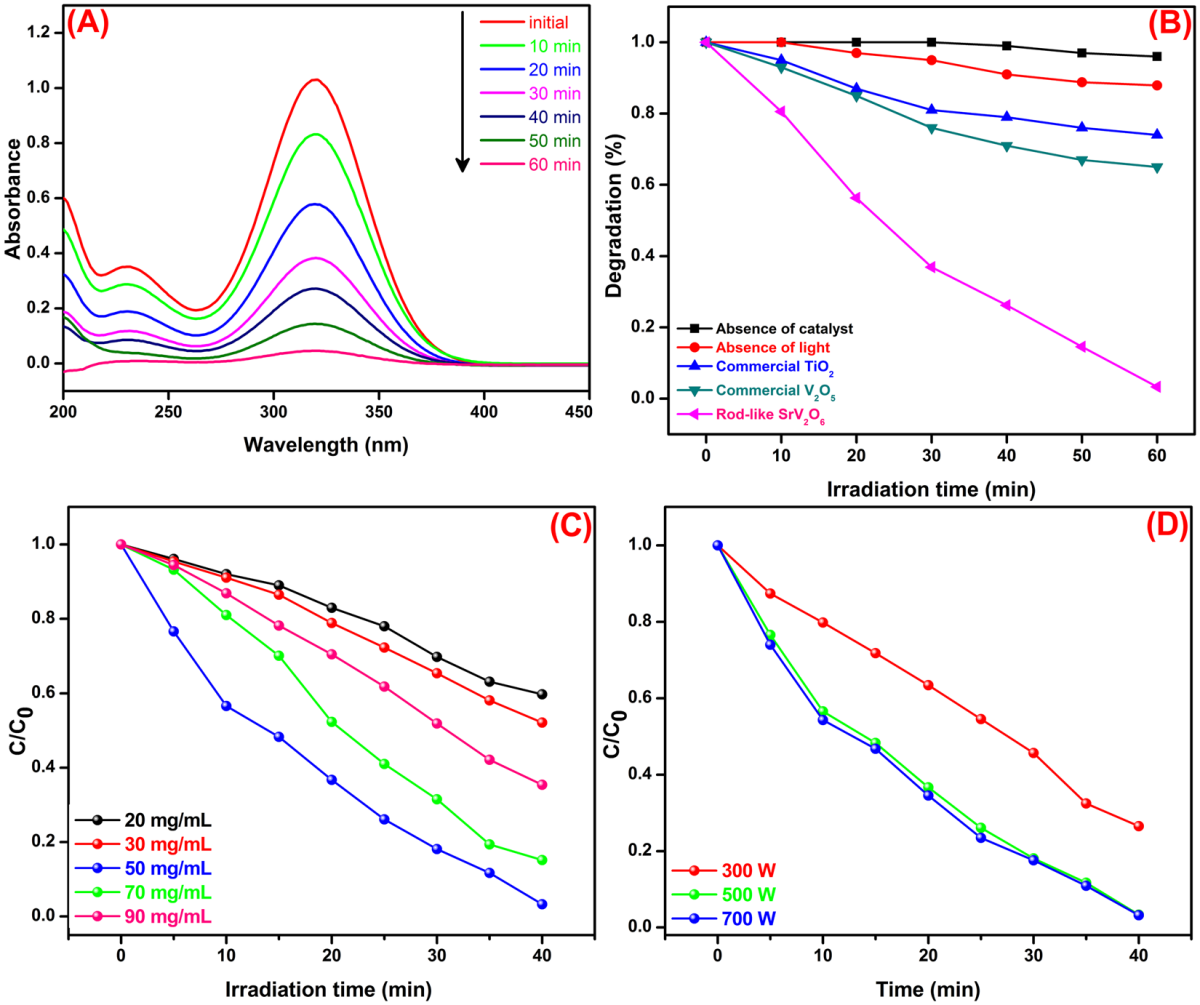
Time-dependent absorption spectrum of MNZ photodegradation (**A**), Effect of different catalyst dosage (**B**), Effect of catalyst dosage and (**C**) Effect of light intensity on the photodegradation of MNZ (**D**).

The photocatalytic performances of rod-like SrV_2_O_6_ were evaluated for the degradation of MNZ in aqueous solution under visible light irradiation. Figure 9A portrays the time-dependent UV-vis absorption spectrum of MNZ solution in the presence of rod-like SrV_2_O_6_ under visible light irradiation. It was observed that, the intensity of major characteristic peak of MNZ at 318 nm gradually diminished with respect to increasing irradiation time, because of generation of electron-hole pairs. After 60 min of irradiation, the absorption intensity of the peak was almost vanished which implies that the complete degradation of MNZ solution. There was no other discernible new peaks were observed which suggests that the intermediates do not absorb the wavelength of 318 nm. Hence, rod-like SrV_2_O_6_ efficiently degraded ~98% of MNZ solution within 60 min under visible light irradiation.

For comparison, we assessed the photocatalytic activity of commercial TiO_2_ and commercial V_2_O_5_ under the same identical conditions. Blank tests were also carried out, irradiation in the absence of photocatalyst and photocatalyst alone without irradiation and the results were presented in Fig. 9B. From the Fig. 9B, there was no significant degradation was observed in the absence of photocatalyst which revealed that MNZ is stable under visible light irradiation. The adsorption of the MNZ solution on the surface of the rod-like SrV_2_O_6_ photocatalyst was also evaluated and the results represents only 12% of MNZ degradation was achieved. Meanwhile, the commercial TiO_2_ and V_2_O_5_ exhibited the degradation of MNZ is 24 and 32% respectively which clearly proved that the as-synthesized rod-like SrV_2_O_6_ possessed superior photocatalytic property against MNZ solution under visible light irradiation.

The photocatalytic degradation efficiency strongly depends on amount of catalyst used in the photocatalytic reaction. Hence, a sequence of experiments was carried out by varying the amount of rod-like SrV_2_O_6_ catalyst dosage from 20 to 90 mg/mL while other parameters are kept same and the results are presented in Fig. 9C. As can be seen, the degradation efficiency was increased with increasing amount of catalyst dosage; the degradation efficiency was very high at 50 mg/mL and thereafter considerably decreased. Though, the degradation efficiency was higher when the amount of catalyst dosage is about 50 mg/mL which might be due to the complete consumption of incident light photons striking on the catalyst surface and/or availability of number of active sites at the surface. Beyond 50 mg/mL, the degradation efficiency was decreased which is due to the aggregation of catalyst particles which reduce the interfacial area between the reaction solution and the catalyst. As a result, a number of active sites that reaches on the surface of the catalyst are decreased. In addition, light scattering and high cloudiness were happened which also reduce photodegradation efficiency.

Photodegradation performances were also depending on the light intensity and therefore we evaluated the effect of light intensity for the photodegradation of MNZ and other parameters are kept constant. Figure 9D, demonstrates the increasing of photodegradation performance with increasing the light intensity from 300 W to 500 W. Beyond that, the degradation performances were maintained at constant. Hence, 500 W light intensity is much enough for the photodegradation of MNZ solution.

**Figure 10:**
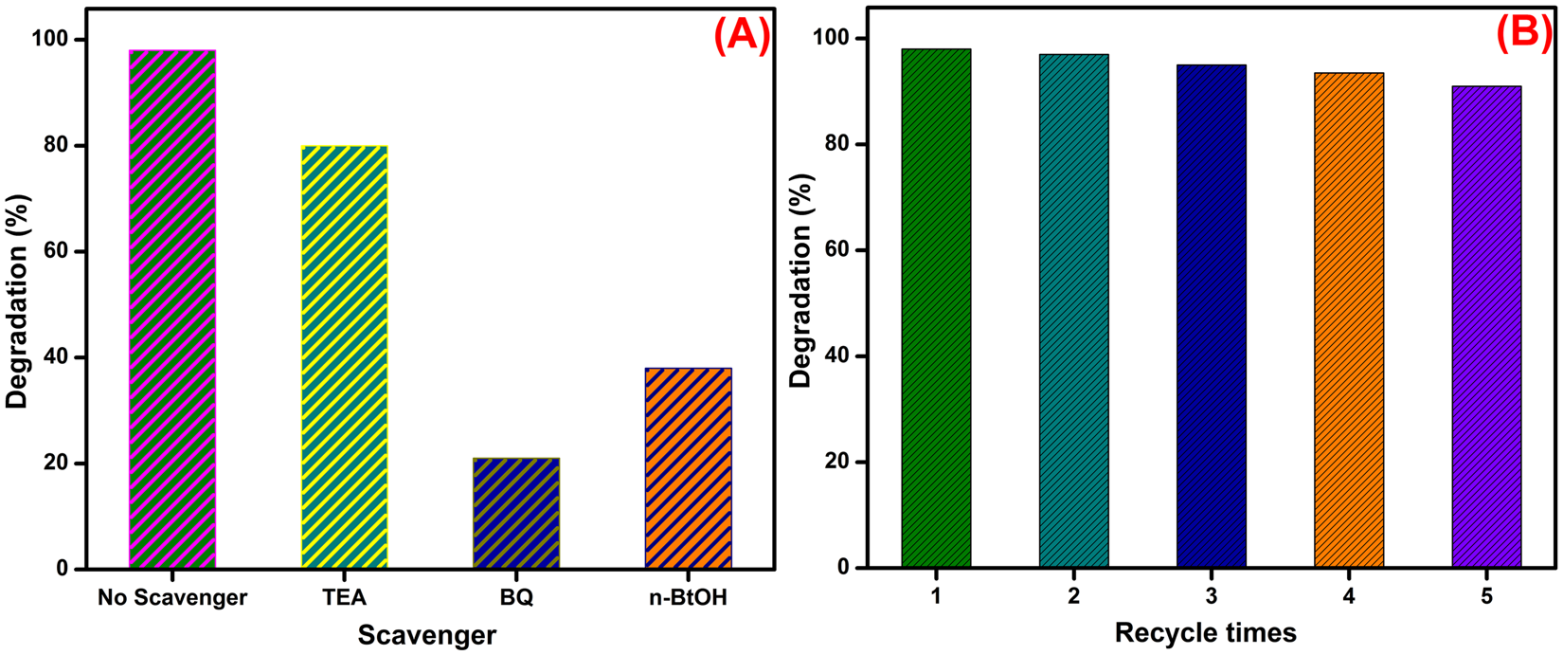
Effect of scavengers on the photodegradation of MNZ and (**A**) Stability of the rod-like SrV_2_O_6_ (**B**).

In order to identify the active involvement of reactive oxidative species (ROS) such as hole (h^+^), superoxide radicals (O_2_^.−^) and hydroxyl radicals (•OH) in the photodegradation process, the scavenger experiments were carried out. In this study, triethylamine (TEA), benzoquinone (BQ) and n-butanol (n-BtOH) were used as the scavengers for h^+^, O_2_^.−^ and •OH species respectively and the results are shown in Fig. 10A. The •OH scavenger, obviously BtOH strongly inhibited that the photocatalytic reaction proceeded by the active involvement of •OH. In the present study, it was observed that, the addition of n-BtOH and BQ was greatly suppressed which clearly revealed that the active involvement of •OH radical. However, the addition of TEA was slightly decreased the photodegradation rate which may be attributed to the active involvement of O_2_^.−^. Hence, the •OH and O_2_^.−^ species plays important responsibility and h^+^ plays a minor role for the degradation of MNZ solution.

### Stability

The stability and reusability of the photocatalyst is also extremely pivotal due to its practical applications. Therefore, we evaluated the recycling experiments were conducted for the rod-like SrV_2_O_6_ photocatalyst over five repeated cycles under the similar conditions and the results presented in Fig. 10B. It can be seen that, after five recycling runs the degradation efficacy was slightly decreased from 98 to 92%. The slight loss is due to the adsorption of MNZ solution on the surface of the photocatalyst which might be inhibited the generation of reactive species and results in fewer numbers. The results showed that the as-synthesized rod-like SrV_2_O_6_ had good stability even after five cycles.

### Plausible photocatalytic mechanism for the degradation of MNZ solution

The reaction pathway of the photocatalytic reaction was an important parameter to determine the stepwise progress of the reaction. The photocatalytic degradation of MNZ was followed by the reaction pathway as given below

1 $$Sr{V}_{2}{O}_{6}+h\nu \to Sr{V}_{2}{O}_{6}({e}_{CB}^{\cdot }+{h}_{VB}^{+})$$

2 $$Sr{V}_{2}{O}_{6}({e}_{CB}^{-})+{O}_{2}\to Sr{V}_{2}{O}_{6}+{O}_{2}^{.-}$$

3 $$Sr{V}_{2}{O}_{6}({h}_{VB}^{+})+{H}_{2}O\to Sr{V}_{2}{O}_{6}+HO\bullet $$

4 $${O}_{2}^{.-}+{H}^{+}\to \bullet H{O}_{2}$$

5 $$HO\bullet ,{O}_{2}^{.-},\bullet H{O}_{2},+MNZ\to MN{Z}^{\bullet +}+{H}_{2}O$$

6 $$HO\bullet ,{O}_{2}^{.-},\bullet H{O}_{2},+MN{Z}^{\bullet +}\to derivatives\,of\,MNZ\to {H}_{2}O+C{O}_{2}$$

Under visible light illumination the rod-like SrV_2_O_6_ were excited and the electrons (e^−^) in the valence band is moved to the conduction band (CB) while leaving a hole in the valance band (VB) as illustrated in eq. 1. The e^−^ in the CB and h + in the VB were reacted with the O_2_ and H_2_O to produced the paramount important O_2_^.−^ (eq. 2) and •OH respectively. Furthermore, O_2_^.−^ was further reacted with H^+^ to form •HO_2_ and the active ROS was degraded the MNZ pollutant.

## Conclusions

In conclusion, rod-like SrV_2_O_6_ were successfully developed by a simple hydrothermal method. The prepared rod-like SrV_2_O_6_ were characterized by XRD, Raman, SEM, EDX, TEM, XPS, BET and UV-DRS. These techniques were clearly confirmed the structural nature and morphology of the SrV_2_O_6._ The entire results are robustly proofed that the as-formed products were pure, without any other impurities. However, the developed rod-like SrV_2_O_6_ explored good electrocatalytic activity for the detection of CA even in the presence of glucose, galactose, fructose, sucrose, catechol, ferulic acid, gallic acid, uric acid, ascorbic acid and dopamine. The flexible electrochemical sensor based on SrV_2_O_6_/GCE is very helpful for determination of CA and has more advantageous properties such as low LOD, good anti-interference ability, good sensitivity and wide linear response range. Moreover, the as-synthesized rod-like SrV_2_O_6_ showed highly efficient and versatile photocatalytic performances for the degradation of MNZ, which degrades above 98% of MNZ solution under visible light irradiation within 60 min. Ambitiously, rod-like SrV_2_O_6_ can be used as advanced electrode material as well as photocatalyst for the detection and degradation of CA and MNZ.

## Experimental Section

### Materials

Strontium nitrate (SrNO_3_), ammonium metavanadate (NH_4_VO_3_), caffeic acid (C_9_H_8_O_4_), glucose, galactose, fructose, sucrose, catechol, ferulic acid, gallic acid, uric acid, ascorbic acid and dopamine were received from Sigma-Aldrich and Alfa Aesar Companies. All other chemicals/reagents are analytical grade were purchased with maximum high purity and used without further purification. All required solution was prepared by using de-ionized (DI) water for the throughout the experiments.

### Synthesis of rod-like SrV_2_O_6_

In a typical synthesis, 0.5 mM of Sr(NO)_3_ and 0.3 mM of NH_4_VO_3_ were dissolved in 80 mL DI ﻿water﻿ under vigorous stirring for 1 h and the solution was transferred into 100 mL Teflon-lined sealed stainless steel autoclave at 180 °C for 8 h. Then, the products were collected by centrifugation and thoroughly washed with copious amount of DI water and dried at 80 °C for 6 h. The dried products were annealed at 500 °C for 2 h before further characterization.

### Characterization

The powder XRD patterns of the product was recorded on a Bruker D8 Advance X-ray diffractometer with monochromatized Cu-Kα radiation (λ = 1.5418 A^0^). An accelerating voltage of 40 kV, the emission current of 40 mA and a step size of 0.02^0^ with a step time of 0.5 secs were used. TEM images were recorded on a JEOL JEM-3010 microscope. SEM was recorded using Hitachi S-3000 H electron microscope. EDX spectrum analyzed using HORIBA EMAX X-ACT that was attached with Hitachi S-3000 H scanning electron microscope. UV-vis diffuse reflectance spectra were carried out on a Lambda 750 (Perkin Elmer) spectrophotometer at a wavelength range of 200–800 nm. The Raman spectra of the sample were measured on a Renishaw 1000 Raman microscope system, the excitation source was a He/Ne laser (λ = 633 nm) and the resolution was 2 cm^−1^. XPS measurements were achieved by a Thermo Scientific ESCA Lab 250 spectrophotometer, and all of the binding energies were calibrated by the C 1 s peak at 284.6 eV. The absorption spectra in the photodegradation procedure were observed by Shimadzu 2100 UV-visible spectrometer. The specific surface area and pore size distribution were determined using the BET (Micromeritics, ASAP 2020 M). All electrochemical measurements were measured using CHI 405a work station in a three conventional electrode system, modified glassy carbon electrode (GCE-surface area 0.07 cm^2^) as a working electrode, Pt wire as a counter electrode and saturated Ag/AgCl (KCl) as a reference electrode. An amperometric (*i-t*) measurement was carried out with analytical rotator AFMSRX (PINE instruments, USA) and rotation disc glassy carbon electrode (RDGCE) with working area 0.2 cm^2^. All the electrochemical measurements were performed at room temperature.

### Fabrication of rod-like SrV_2_O_6_ on the GCE surface for CA

To fabricate the CA sensor, the GCE was mirror-like polished with 0.05 µm alumina slurry and thoroughly washed with copious amount of DI water. After that, 5 mg/mL of as-prepared rod-like SrV_2_O_6_ was redispersed in DI water and sonicated on hour to get the homogeneous suspension. The rod-like SrV_2_O_6_ modified GCE was fabricated by a simple drop casting method. An 8 µL suspension of re-dispersed SrV_2_O_6_ was drop coated on the GCE surface and it was kept in air oven at ambient temperature. Then, the dried modified GCE was gently washed with DI water to remove the loosely attached molecules on the GCE surface. Then that obtained SrV_2_O_6_ modified GCE was used to further electrochemical measurements.

### Photocatalytic experiments

The photocatalytic activity of the as-prepared rod-like SrV_2_O_6_ was evaluated for the degradation of MNZ aqueous solution under visible light irradiation. In a typical experiment, 50 mg of catalyst was dispersed in 200 mL aqueous solutions of MNZ (20 mg/L). Prior to illumination, the solution mixture was stirred magnetically for 30 min in the dark to ensure that the MNZ attained absorption–desorption equilibrium on the photocatalyst surface. A 500 W tungsten lamp equipped with a UV cut-off filter (λ > 400 nm) was used as the visible light source for the photodegradation reactions and the experimental set up is presented in Fig. S2. At defined irradiation intervals, the aliquots (5.0 mL) of the suspension were collected and centrifuged to separate the photocatalyst particles. The supernatant solution was analyzed by UV-vis spectrophotometer to determine the concentration changes of MNZ. The photocatalytic degradation efficiency was calculated using C/C_0_, where C is the concentration of MNZ solution at certain irradiation time (t) while C_0_ is the adsorption/desorption equilibrium concentration of MNZ (t = 0).

## Electronic supplementary material

Supplementary Information

## References

[1] Balasundram N, Sundram K, Samman S (2006). Phenolic compounds in plants and agri-industrial by-products: Antioxidant activity, occurrence, and potential uses. Food Chem..

[2] Salcedo CL, Nazareno MA (2015). Effect of phenolic compounds on the oxidative stability of ground walnuts and almonds. RSC Adv..

[3] Arnous A, Makris DP, Kefalas P (2001). Effect of principal polyphenolic components in relation to antioxidant characteristics of aged red wines. J. Agric. Food Chem..

[4] Zheng B (2014). Green preparation of reduced graphene oxide for sensing and energy storage applications. Sci. Rep..

[5] Tyszczuk K, Skalska-Kaminska A, Wozniak A (2011). Voltammetric method using a lead film electrode for the determination of caffeic acid in a plant material. Food Chem..

[6] Huang MT, Ferraro T (1992). Phenolic-compounds in food and cancer prevention. ACS Symp. Ser..

[7] Robinson WE, Reinecke MG, Abdel-Malek S, Jia Q, Chow SA (1996). Inhibitors of HIV-1 replication that inhibit HIV integrase. Proc. Natl. Acad. Sci. USA.

[8] Cai N, Li Y, Chen S, Su X (2016). Fluorometric Assay Platform for Caffeic Acid Detection Based on the G-quadruplex/hemin DNAzyme. Analyst..

[9] Fracassetti D (2011). Quantification of glutathione, catechin and caffeic acid in grape juice and wine by a novel ultra-performance liquid chromatography method. Food Chem..

[10] Robbins RJ (2003). Phenolic acids in foods: an overview of analytical methodology. J. Agric. Food Chem..

[11] Zitka O (2011). Comparison of Various Easy-to-Use Procedures for Extraction of Phenols from Apricot Fruits. Molecules..

[12] Peng YY, Liu FH, Ye JN (2005). Determination of phenolic acids and flavones in lonicera japonica thumb. by capillary electrophoresis with electrochemical detection. Electroanalysis.

[13] Karthik R (2016). Eco-friendly synthesis of Ag-NPs using Cerasus serrulata plant extract–Its catalytic, electrochemical reduction of 4-NPh and antibacterial activity. J. Ind. Eng. Chem..

[14] Karthik R (2017). A Study of Electrocatalytic and Photocatalytic Activity of Cerium Molybdate Nanocubes Decorated Graphene Oxide for the Sensing and Degradation of Antibiotic Drug: Chloramphenicol. ACS Appl. Mater. Interfaces..

[15] Karikalan N, Karthik R, Chen SM, Velmurugana M, Karuppiah C (2016). Electrochemical properties of the acetaminophen on the screen printed carbon electrode towards the high performance practical sensor applications. J. Colloid Interface Sci..

[16] Dong S (2014). Facile synthesis of novel ZnO/RGO hybrid nanocomposites with enhanced catalytic performance for visible light-driven photodegradation of metronidazole. Mater. Chem. Phys..

[17] Farzadkia M, Esrafili A, Baghapour MA, Shahamat YD, Okhovat N (2013). Degradation of metronidazole in aqueous solution by nano-ZnO/UV photocatalytic process. Desalin. Water Treat..

[18] Bendesky A, Menendez D, Ostrosky-Wegman P (2002). Is metronidazole carcinogenic. Mutat Res., Rev. Mutat. Res..

[19] Saidi I (2014). Indirect electroreduction as pretreatment to enhance biodegradability of metronidazole. J. Hazard. Mater..

[20] Richardson ML, Bowron JM (1985). The fate of pharmaceutical chemicals in the aquatic environment. J. Pharm. Pharmacol..

[21] Rabolle M, Spliid NH (2000). Sorption and mobility of metronidazole, olaquindox, oxytetracycline and tylosin in soil. Chemosphere..

[22] Farzadkia M, Bazrafshan E, Esrafili A, Yang J, Siboni MS (2015). Photocatalytic degradation of Metronidazole with illuminated TiO_2_ nanoparticles. J. Environ. Health Sci. Eng..

[23] Kumar JV, Karthik R, Chen SM, Muthuraj V, Chelladurai K (2016). Fabrication of potato-like silver molybdate microstructures for photocatalytic degradation of chronic toxicity ciprofloxacin and highly selective electrochemical detection of H_2_O_2_. Sci. Rep..

[24] Dong. S (2014). Shape-controlled synthesis of BiVO_4_ hierarchical structures with unique natural-sunlight-driven photocatalytic activity. Appl. Catal. B..

[25] Dong. S (2014). Designing three-dimensional acicular sheaf shaped BiVO_4_/reduced graphene oxide composites for efficient sunlight-driven photocatalytic degradation of dye wastewater. Chem. Eng. J..

[26] Dong. S (2017). Self-assembled hollow sphere shaped Bi_2_WO_6_/RGO composites for efficient sunlight-driven photocatalytic degradation of organic pollutants. Chem. Eng. J..

[27] Dong. S (2014). ZnSnO_3_ hollow nanospheres/reduced graphene oxide nanocomposites as high-performance photocatalysts for degradation of metronidazole. Appl. Catal. B..

[28] Dong. S (2016). Solar photocatalytic degradation of sulfanilamide by BiOCl/reduced graphene oxide nanocomposites: Mechanism and degradation pathways. J. Alloys Compd..

[29] Hu. L (2015). Effects of sodium dodecyl benzene sulfonate on the crystal structures and photocatalytic performance of ZnO powders prepared by hydrothermal method. J. Alloys Compd..

[30] Dong. S (2015). Recent developments in heterogeneous photocatalytic water treatment using visible-light-responsive photocatalysts: A review. RSC Adv..

[31] Ong. WJ (2014). Facet-Dependent Photocatalytic Properties of TiO_2_-Based Composites for Energy Conversion and Environmental Remediation. ChemSusChem.

[32] Ong. WJ (2014). Enhanced daylight-induced photocatalytic activity of solvent exfoliated graphene (SEG)/ZnO hybrid nanocomposites towards degradation of Reactive Black 5. Ind. Eng. Chem. Res..

[33] Ong. WJ (2014). Synergistic effect of graphene as a co-catalyst for enhanced daylight-induced photocatalytic activity of Zn_0.5_Cd_0.5_S synthesized via an improved one-pot co-precipitation-hydrothermal strategy. RSC Adv..

[34] Nithya VD, Selvan RK, Sanjeeviraja C, Radheep DM, Arumugam S (2011). Synthesis and characterization of FeVO_4_ nanoparticles. Mater. Res. Bull..

[35] Zhou Q, Shao M, Chen T, Xu H (2010). Strontium vanadate nanoribbons: Synthesis, characterization and detection of dopamine. Mater. Res. Bull..

[36] Vignesh K, Hariharan R, Rajarajan M, Suganthi A (2013). Visible light assisted photocatalytic activity of TiO_2_–metal vanadate (M = Sr, Ag and Cd) nanocomposites. Mater. Sci. Semicond. Process..

[37] Macias J, Yaremchenko AA, Frade JR (2014). Redox transitions in strontium vanadates: Electrical conductivity and dimensional changes. J Alloys Compd..

[38] Sawala NS, Bajaj NS, Omanwar SK (2016). Near-infrared quantum cutting in Yb3+ ion doped strontium vanadate. Infrared Phys Techn..

[39] Li Y (2015). Pressure-induced amorphization of metavanadate crystals SrV_2_O_6_ and BaV_2_O_6_. J. Appl. Phys..

[40] Li Z, Yang H, Tian HF, Zhang Y, Li JQ (2007). Fabrication and Characterization of Micro-Pattern Dandelion-like and Nanobelts of β-SrV_2_O_6_ via Hydrothermal Process. Chin. J. Chem. Phys..

[41] Yu J, Dai G, Cheng B (2010). Effect of Crystallization Methods on Morphology and Photocatalytic Activity of Anodized TiO2 Nanotube Array Films. J. Phys. Chem. C..

[42] Liu S (2015). Foamed single-crystalline anatase nanocrystals exhibiting enhanced photocatalytic activity. J. Mater. Chem. A..

[43] Wang ZL (2000). Characterizing the Structure and Properties of Individual Wire-Like Nanoentities. Adv. Mater..

[44] Shen ZX, Ong CW, Tang SH, Kuok MH (1994). Spectroscopic evidence of pressure-induced amorphization in α-NaVO_3_. Phys. Rev..

[45] Seetharaman S, Bhat HL, Narayanan PS (1983). Raman spectroscopic studies on sodium metavanadate. J. Raman Spectrosc..

[46] Yan Y, Yu Y, Wu D, Yang Y, Cao Y (2016). TiO_2_/vanadate (Sr_10_V_6_O_25_, Ni_3_V_2_O_8_, Zn_2_V_2_O_7_) heterostructured photocatalysts with enhanced photocatalytic activity for photoreduction of CO_2_ into CH_4_. Nanoscale.

[47] Ai-ju X, Zhaorigtu B, Mei-lin J, Lin Q (2007). Study on Performance of Ni_3_V_2_O_8_ Catalyst and Analysis of X-Ray Photoelectron Spectroscopy. Spectros. Spect. Anal..

[48] Lei Y (2014). Rapid microwave-assisted green synthesis of 3D hierarchical flower-shaped NiCo_2_O_4_ microsphere for high-performance supercapacitor. ACS Appl. Mater. Interface.

[49] Santos DP, Bergamini MF, Fogg AG, Zanoni MVB (2005). Application of a Glassy Carbon Electrode Modified with Poly(Glutamic Acid) in Caffeic Acid Determination. Microchim. Acta.

[50] Fernandes SC, Oliveira IRWZ, Vieira IC (2007). A green bean homogenate immobilized on chemically crosslinked chitin for determination of caffeic acid in white wine. Enzyme Microb. Tech..

[51] Silva LF, Stradiotto NR, Oliveira HP (2008). Determination of Caffeic Acid in Red Wine by Voltammetric Method. Electroanalysis..

[52] Carlo D (2012). Green synthesis of gold–chitosan nanocomposites for caffeic acid sensing. Langmuir..

[53] Zhang Y (2013). Electrochemical Behavior of Caffeic Acid Assayed with Gold Nanoparticles/Graphene Nanosheets Modified Glassy Carbon Electrode. Electroanal..

[54] Filik H (2013). Square-wave stripping voltammetric determination of caffeic acid on electrochemically reduced graphene oxide–Nafion composite film. Talanta..

[55] Leite FRF, Santos WJR, Kubota LT (2014). Selective determination of caffeic acid in wines with electrochemical sensor based on molecularly imprinted siloxanes. Sensor. Actuat. B-Chem..

[56] Kahl M, Golden TD (2014). Electrochemical Determination of Phenolic Acids at a Zn/Al Layered Double Hydroxide Film Modified Glassy Carbon Electrode. Electroanal..

[57] David IG (2015). Rapid determination of total polyphenolic content in tea samples based on caffeic acid voltammetric behaviour on a disposable graphite electrode. Food Chem..

[58] Hongjiao Z (2016). Highly Sensitive Determination of Caffeic Acid Using a Multiwalled Carbon Nanotubes Modified Electrode with NButylpyridinium Hexafluorophosphate Ionic Liquid and Chitosan as Binders. Int. J. Electrochem. Sci..

[59] Ibarra-Escutia P, Gomez JJ, Calas-Blanchard C, Marty JL, Ramirez-Silva MT (2010). Amperometric biosensor based on a high resolution photopolymer deposited onto a screen-printed electrode for phenolic compounds monitoring in tea infusions. Talanta..

[60] Diaconu M, Simona C, Litescu, Gabriel LR (2010). Laccase–MWCNT–chitosan biosensor-A new tool for total polyphenolic content evaluation from *in vitro* cultivated plants. Sens. Actuators B-chem..

[61] Cortina-Puig M, Murioz-Berbel X, Calas-Blanchard C, Marty JL (2010). Diazonium-functionalized tyrosinase-based biosensor for the detection of tea polyphenols. Microchim. Acta..

[62] Karabiberoğlu ŞU, Ayan EM, Dursun Z (2013). Investigation of thermodynamic parameters using an Ag nanoparticles modified poly (thiophene) film glassy carbon electrode. Electroanalysis..

[63] Kaoutit M (2008). E. et. A comparison of three amperometric phenoloxidase–Sonogel–Carbon based biosensors for determination of polyphenols in beers. J. Cisneros, Food Chem..

[64] Magarelli G (2013). Development and validation of a voltammetric method for determination of total phenolic acids in cotton cultivars. Microchem. J..

[65] Blasco AJ, Gonz.lez MC, Escarpa A (2004). Electrochemical approach for discriminating and measuring predominant flavonoids and phenolic acids using differential pulse voltammetry: towards an electrochemical index of natural antioxidants. Anal. Chim. Acta..

