# Supplementary material for: A selective electrochemical sensor for caffeic acid and photocatalyst for metronidazole drug pollutant - A dual role by rod-like SrV2O6
Source: Sci Rep. 2017 Aug 3;7:7254. doi: 10.1038/s41598-017-07423-1 (PMC5543073; doi:10.1038/s41598-017-07423-1)
Supplement: Supplementary file 1 — Supplementary Information [file 41598_2017_7423_MOESM1_ESM.pdf]

A selective electrochemical sensor for caffeic acid and photocatalyst for metronidazole drug pollutant - A dual role by rod-like SrV_2O_6

R.Karthik[1], J.Vinoth Kumar[2], Shen-Ming Chen[1*], P. Senthil Kumar[2, 3], V. Selvam[2], V. Muthuraj[2*]

[1]Department of Chemical Engineering, National Taipei University of Technology, No. 1, Section 3, Chung-Hsiao East Road, Taipei 106, Taiwan, ROC.

[2]Department of Chemistry, VHNSN College, Virudhunagar – 626001, Tamilnadu, India**.**

[3]Department of Chemistry, International Research Center, Kalasalingam University, Krishnankoil - 626126, Virudhunagar, Tamilnadu, India

*** Authors for Correspondence**

E-mail: smchen78@ms15.hinet.net, Tel: +886 2270 17147, Fax: +886 2270 25238.

E-mail: muthuraj75@gmail.com, Tel: +919940965228

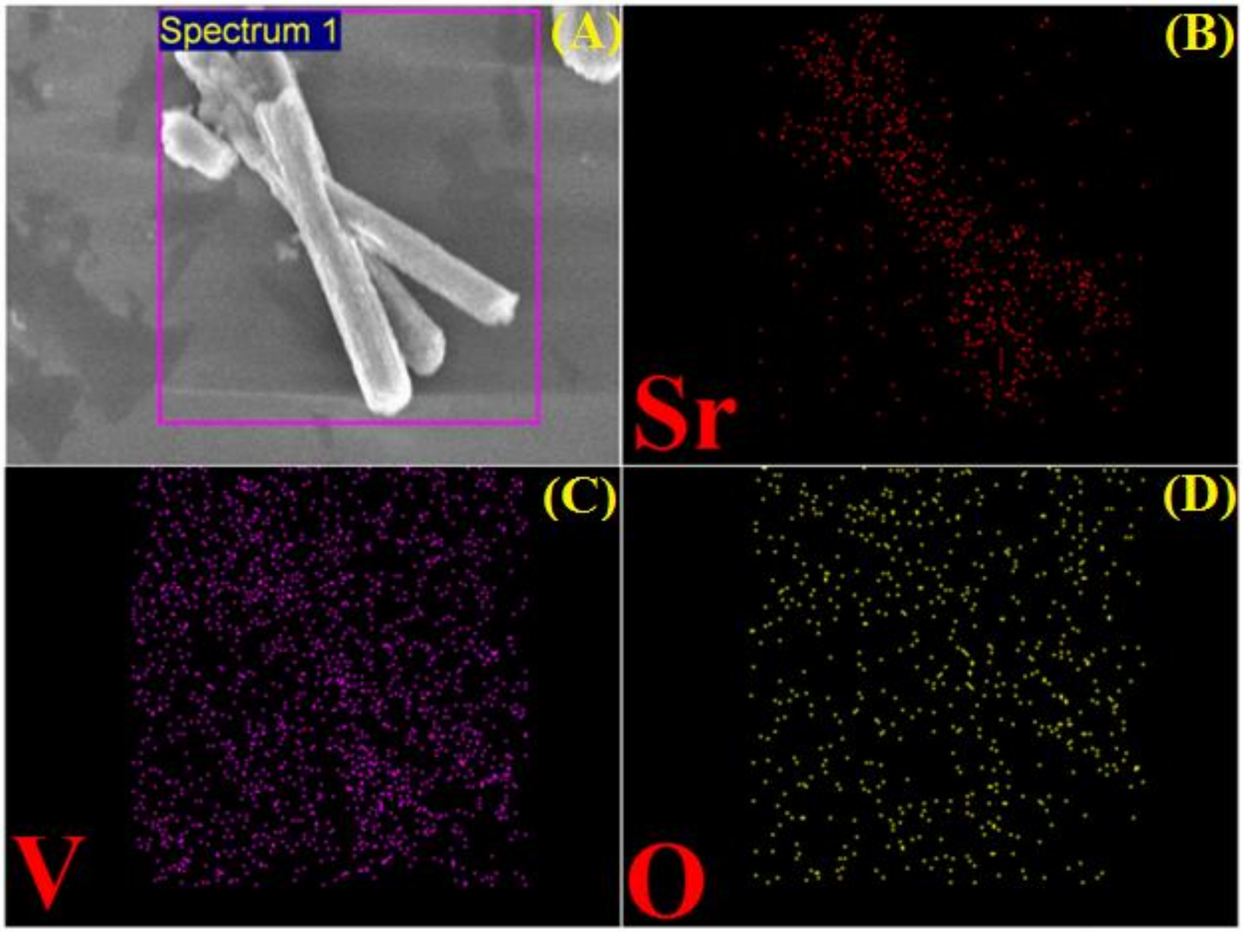

Fig. *S1*. SEM images of rod-like SrV_2O_6 (A) and overall EDX mapping of SrV_2O_6 for Strontium (B) Vanadium (C) and Oxygen (D).

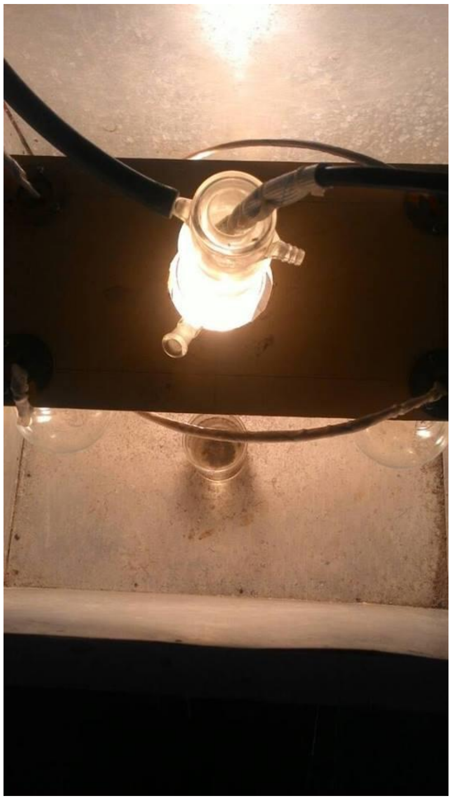

Fig. *S2*. Experimental set up for the photodegradation of MNZ over rod-like SrV_2O_6.
